# Advances in Polyoxometalates as Electron Mediators for Photocatalytic Dye Degradation

**DOI:** 10.3390/ijms242015244

**Published:** 2023-10-17

**Authors:** Ruyue Li, Yaqi Wang, Fei Zeng, Cuiqing Si, Dan Zhang, Wenbiao Xu, Junyou Shi

**Affiliations:** Key Laboratory of Biomass Materials Science and Technology of Jilin Province, Beihua University, Binjiang East Road, Jilin 132013, China; 17852762617@163.com (R.L.); yqwang0929@163.com (Y.W.); feiscientist@163.com (F.Z.); qq2598248103@163.com (C.S.); wenbiao.xu@beihua.edu.cn (W.X.)

**Keywords:** polyoxometalates, dyes, photodegradation

## Abstract

The increasing concerns over the environment and the growing demand for sustainable water treatment technologies have sparked substantial interest in the field of photocatalytic dye removal. Polyoxometalates (POMs), known for their intricate metal–oxygen anion clusters, have received considerable attention due to their versatile structures, compositions, and efficient facilitation of photo-induced electron transfers. This paper provides an overview of the ongoing research progress in the realm of photocatalytic dye degradation utilizing POMs and their derivatives. The details encompass the compositions of catalysts, catalytic efficacy, and light absorption propensities, and the photocatalytic mechanisms inherent to POM-based materials for dye degradation are exhaustively expounded upon. This review not only contributes to a better understanding of the potential of POM-based materials in photocatalytic dye degradation, but also presents the advancements and future prospects in this domain of environmental remediation.

## 1. Introduction

As chemical entities, dyes are used in various industries such as textile manufacturing [1], leather production [2], papermaking [3], cosmetics [4,5], and food processing [6], playing an indispensable role in modern industrial processes and daily life. However, the rapid expansion of the textile printing and dyeing sector has led to a significant increase in the release of dye-laden wastewater, exacerbating water pollution [7]. The presence of aromatic rings, azo groups, and other chemical moieties within these dyes renders them particularly detrimental to the well-being of organisms [1,8]. Furthermore, dyes exhibit remarkable stability and solubility, thereby necessitating intricate, multi-stage treatment strategies for effective degradation [9].

Dyes are commonly classified into three distinct categories based on their structural configurations: azo, anthraquinone, and heterocyclic [10,11]. Azo dyes, exemplified by methyl orange (MO) [12], methyl blue [13], and Congo red (CR) [14], are extensively used in the synthesis of colorants within the textile industry. Anthraquinone dyes, characterized by vibrant and diverse color profiles, include red 3B [15], reduced yellow G (yellow anthraquinone), and reduced blue RS (blue anthraquinone). Heterocyclic dyes include both oxygen and nitrogen heterocyclic rings [16], encompassing examples like rhodamine B (RhB) [17] and methylene blue (MB). Moreover, charges can serve as an effective criterion for classifying dyes. This leads to the categorization of dyes into three primary classes: cationic dyes, anionic dyes, and nonionic dyes [18]. Cationic dyes (e.g., MB and RhB) carry positive charges and are conventionally applied to negatively charged fibers, such as cotton and linen [19]. The dyeing process with these dyes involves interactions with the fibers’ inherent negative charges. Conversely, anionic dyes (e.g., MO and CR) exhibit negative charges and find utility in positively charged fibers like wool and silk, interacting with the fibers’ positive charge during dyeing [20,21]. And nonionic dyes, exemplified by the Cibacron series, are devoid of charges, and their dyeing relies on nonionic interactions, such as hydrogen bonding [22]. Among the array of dyes, MB, RhB and MO are the frequently employed options. However, exposure to and the consumption or ingestion of these dyes could potentially result in harm to organisms [23]. Upon entering the body, MO undergoes metabolism by intestinal microorganisms into aromatic amines, involved in the initiation of intestinal cancer [24,25]. MB has the propensity to accumulate within the body through water or food chains, even at trace levels, potentially inducing carcinogenic and mutagenic effects [26,27,28]. Medically, RhB has been definitively shown to be neurotoxic and carcinogenic, capable of irritating the skin, respiratory tract, and eyes [29]. Consequently, the imperative removal of dyes from water holds substantial significance, as it is pivotal for the preservation of organismal health and the attainment of sustainable development.

Photocatalytic technology replicates natural photosynthesis, utilizing light as the energy source to activate the redox capabilities of photocatalysts across diverse light conditions. Under light irradiation, photocatalysts absorb energy, promoting the transition of electrons to higher energy levels and generating electron–hole pairs. These pairs then react with oxygen and water to produce active species like O_2_^•−^ and ^•^OH. These active species work in tandem with electron–hole pairs to convert pollutants into water and carbon dioxide [30,31]. In comparison to conventional dye removal techniques, photocatalysis exhibits distinct advantages, characterized by its mild reaction conditions, environmental compatibility, non-toxic nature, and cost-effectiveness [30]. Despite the drawbacks of light dependence and the necessity for specific catalytic materials, photocatalysis retains its potential as a green and clean technology in water treatment and environmental management [32].

Polyoxometalates (POMs) are compounds consisting of metal–oxo clusters, wherein early transition metals (such as Mo, W, V, etc.) are connected via bridging oxygen linkages [33,34,35]. The amalgamation of various metals imparts POMs with a range of electronic structures, enabling them to absorb light across multiple wavelengths and display photocatalytic powers [36,37,38]. When incident light energy surpasses the bandgap energy, a process of electron excitation transpires within POMs, propelling electrons from the ground state (valence band) to the excited state (conduction band) [39]. Particularly, electrons of oxygen atom within metal–oxygen clusters become excited, subsequently transiting to the conduction band (CB) of metals and occupying the vacant d orbitals of metals in metal–oxygen clusters. This transition encompasses the shift from the highest occupied molecular orbital (HOMO) of O^2−^ to the lowest unoccupied molecular orbital (LUMO) associated with W^6+^/Mo^6+^/V^5+^ [40,41,42]. This progression generates electron–hole pairs conducive to pollutant oxidation or radical generation [43]. Additionally, the interplay among distinct metals and the formation of oxygen cluster structures within POMs yield expanded reaction pathways [44,45,46]. Furthermore, the fine-tuning of POMs’composition and structure facilitates the creation of precise electronic configurations, aligning the material’s bandgap with the energy spectrum of incident light, thereby heightening photocatalytic efficiency [42].

Endowed with robust photocatalytic activity facilitated by efficient charge separation capabilities, POMs emerge as a promising avenue for sustainable water treatment [47,48,49,50]. However, a notable gap exists in the documentation of POMs’ effectiveness and mechanisms in dye removal from aqueous solutions. By scrutinizing the current research, this study has designated MO, RhB, and MB as representative dyes, and the composition of catalysts, removal efficiency, and photocatalytic mechanisms involving POM-based catalysts in connection with these dyes are critically investigated. The objective is to furnish an interdisciplinary comprehension of their potential in confronting contemporary challenges associated with water pollution. Through a systematic review of existing literature, this study aims to provide valuable references for the design of POM-based photocatalysts and offer insights into sustainable strategies for the remediation of dye-contaminated wastewater using POM-based photocatalytic materials.

## 2. Photocatalytic Degradation of Different Dyes with POM-Based Materials

### 2.1. Methylene Blue (MB)

Keggin-type polyanions [XM_12_O_40_]^n−^ (X = B, P, Si, Ge, As; M = W, Mo, V), as one of the fundamental polyanions, possess oxygen-rich surfaces and robust coordination capabilities [51]. Vanadium atoms establish connections with the framework through metal–oxygen bonds, giving rise to vanadium-terminated or vanadium-substituted structures, thereby enhancing the compositional and property diversity of Keggin-type POMs [52]. Fengrui Li et al. [53] obtained a tri-vanadium-capped phosphomolybdate ([Na_3_PMo_12_V_3_O_43_]·4H_2_O, denoted as PMo_12_V_3_) through a hydrothermal reaction for the removal of MB. This compound exhibited distinct characteristic absorption peaks of POMs at 227 nm (O→Mo) and 280 nm (Mo→O→Mo). Under UV irradiation, it achieved a remarkable 99.3% MB removal within 65 min using 500 mg/L PMo_12_V_3_ and 10 mg/L MB, and the removal efficiency remained unchanged after five cycles (Table 1). The photocatalytic performance of PMo_12_V_3_O_43_^3−^ was amplified through vanadium substitution, leading to a reduced band gap of 2.8 eV. When subjected to UV radiation, electron transfer occurred from the HOMO of O to the LUMO of both molybdenum (Mo) and vanadium (V). This process propelled PMo_12_V_3_O_43_^3−^ into an excited state, generating pairs of electrons (e^−^) and holes (h^+^) (Equation (1)) [53]. Concurrently, vanadium facilitated the dispersion of photo-generated electrons owing to its heightened redox characteristics and facilitated electron transfer [54]. In this sequence, photogenerated electrons, functioning as reducing agents, engaged with O_2_ to generate superoxide radicals (O_2_^•−^) (Equation (2)), while photo-generated holes served as oxidants or combined with hydroxides/water to yield hydroxyl radicals (^•^OH) (Equation (3)) [55]. The collective action of O_2_^•−^, ^•^OH, and hole–electron pairs contributed to the removal of MB (Equation (4)).

[Mo^6+^ − O^2−^ − V^5+^] → [Mo^6+^ − O^2−^ − V^4+^]*/[Mo^5+^ − O^2−^ − V^5+^]* (h^+^ + e^−^) (1)

e^−^ + O_2_ → O_2_^•−^ (2)

h^+^ + H_2_O → ^•^OH + H^+^ (3)

MB + h^+^, ^•^OH, O_2_^•−^→ degradation intermediates → CO_2_ + H_2_O (4)

The enhancement of photocatalytic properties can be achieved through the immobilization of POMs onto specific supports. The Keggin-type POM (NH_4_)_4_[PMo_11_VO_40_] was immobilized onto the surface of g-C_3_N_4_ through the dipping technique, resulting in the fabrication of (NH_4_)_4_[PMo_11_VO_40_]/g-C_3_N_4_ (PMo_11_V/g-C_3_N_4_) [56]. In comparison to g-C_3_N_4_, the band intensity of PMo_11_V/g-C_3_N_4_ exhibited an extension from 200 to 500 nm to 200 to 580 nm. Notably, when exposed to visible light irradiation, the removal efficiency reached 94.7% within 120 min using 400 mg/L of PMo_11_V/g-C_3_N_4_ and 10 mg/L of MB (Table 1). In contrast, the corresponding removal efficiency of MB were 29.7% for single g-C_3_N_4_ and 50.8% for (NH_4_)_4_[PMo_11_VO_40_] under visible light irradiation. Benefitting from the favorable electronic properties of (NH_4_)_4_[PMo_11_VO_40_], the combination of (NH_4_)_4_[PMo_11_VO_40_] with g-C_3_N_4_ not only facilitated the generation of holes and electrons but also contributed to the attenuation of hole–electron recombination [56]. This synergy was pivotal in the formation and utilization of h^+^, e^−^, ^•^OH, and O_2_^•−^, thus facilitating the efficient degradation of MB. Similarly, the incorporation of P_2_W_18_Sn_3_ into Nd-TiO_2_ through one-pot synthesis induced a blue shift in absorption, intensifying the ultraviolet absorption capacity [57]. Impressively, a mere 5 min exposure to UV light resulted in 91.0% MB removal (10 mg/L) when subjected to P_2_W_18_Sn_3_/Nd-TiO_2_ (Table 1) [57]. Although both PMo_11_V/g-C_3_N_4_ [56] and P_2_W_18_Sn_3_/Nd-TiO_2_ [57] have achieved high removal efficiencies, there was no indication of stability.

**Figure 1:**
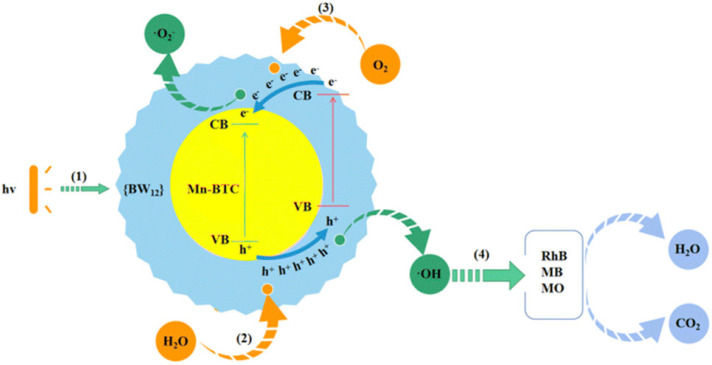
The degradation mechanism of MB with Mn-BTC@Ag_5_[BW_12_] ((1) represented the action of light on the photocatalyst; (2) represented the reaction of water with h^+^ in the photocatalyst to produce ^•^OH; (3) represented the reaction of O_2_ with e^−^ in the photocatalyst to produce O_2_^•−^; (4) represents the generated ^•^OH acting on the substrate). Adapted with permission from Ref. [62].

POMOFs, formed by integrating POMs into metal–organic frameworks (MOFs), not only embody the versatile catalytic traits intrinsic to POMs but also encompass the amplified porosity, expansive surface area, and modifiable pore dimensions characteristic of MOFs [58,59,60,61]. Mn-BTC@Ag_5_[BW_12_O_40_] (Mn-BTC@Ag_5_[BW_12_]; BTC: 1,3,5-benzenetricarboxylic acid) was synthesized using the grinding method, resulting in a core-shell structure [62]. It achieved 95.6% MB removal within 140 min, utilizing 500 mg/L of Mn-BTC@Ag_5_[BW_12_] and 15 mg/L of MB under UV irradiation. Notably, even after undergoing five cycles, the removal efficiency of MB remained unchanged (Table 1) [62]. Upon exposure to UV light, electrons transitioned from the valence band (VB) of both Mn-BTC and Ag_5_[BW_12_] to their conduction band (CB), resulting in the creation of electron–hole pairs (Figure 1) [63]. Additionally, photogenerated holes migrated from the VB of Mn-BTC to that of BW_12_ due to the latter′s more positive VB potential. Furthermore, electron migration occurred from the CB of BW_12_ to that of Mn-BTC, which was facilitated by Mn-BTC’s more negative potential [64]. This process induced the separation of energy levels and reduced the electron transfer time [65]. Among the generated active species, ^•^OH played a pivotal role in the degradation (Figure 1). Using the same synthesis method as Mn-BTC@Ag_5_[BW_12_], Ag_5_[BW_12_]@[Ag_3_(µ-HBTC)(µ-H_2_BTC)]_n_ (termed Ag-BTC@Ag_5_[BW_12_]) [66] and [Zn_4_(BTC)_2_(μ_4_-O)(H_2_O)_2_]@Ag_5_[BW_12_] (denoted as Zn-BTC@Ag_5_[BW_12_]) [67] were obtained with a core–shell structure. Ag-BTC@Ag_5_[BW_12_] achieved a 94.4% MB removal [66] and Zn-BTC@Ag_5_[BW_12_] [67] yielded a 96.1% removal of MB under the same conditions compared to that of Mn-BTC@Ag_5_[BW_12_] (Table 1)**.** Both catalysts demonstrated outstanding activity and stability (Table 1). The 3D host–guest POMOF ([Ag_5_(pz)_6_(H_2_O)_4_[BW_12_], denoted as [Ag_5_(pz)_6_][BW_12_]) was synthesized through hydrothermal reaction for MB removal [68]. Under UV irradiation, using 500 mg/L of [Ag_5_(pz)_6_][BW_12_] and 15 mg/L of MB, 93.2% of the MB was removed in 140 min (Table 1), and the removal efficiency was almost unchanged after five cycles [68]. Due to the charge transfer absorption occurring from oxygen to metals within the shorter wavelength range, the photoresponsive attributes of POMs predominantly manifest in the ultraviolet range (λ = 200−400 nm) [40]. This limitation has restricted their utility in solar-driven photocatalysis [69,70]. However, by coupling with visible light-absorbing materials, an avenue is established to enhance the visible light absorption of POMs [71,72,73]. [Hpip]_2_._5_[BW_12_], formed through inserting BW_12_O_40_^5−^ clusters into the cavities of Hpip groups through heating reflux synthesis, removed 97.1% of MB (20 mg/L) within 24 min under visible light irradiation, which decreased to 90.0% after five cycles (Table 1) [74]. In addition to BW_12_O_40_^5−^, the As^III^-capped Keggin arsenomolybdate with four V^4+^ substitutions ([As_2_^III^As^V^Mo_8_^VI^V_4_^IV^O_40_]^5−^) was also used as the template to produce the POMOF ([As_2_^III^As^V^Mo_8_^VI^V_4_^IV^O_40_]_2_[Cu^I^Cu_2_^II^(pz)_4_]_2_, denoted as [Cu_3_(pz)_4_]_2_[As_3_Mo_8_V_4_]_2_; pz = pyrazine) by hydrothermal method [75]. [Cu_3_(pz)_4_]_2_[As_3_Mo_8_V_4_]_2_ achieved 97.8% removal efficiency within 120 min using 400 mg/L of [Cu_3_(pz)_4_]_2_[As_3_Mo_8_V_4_]_2_ and 10 mg/L of MB under UV irradiation (Table 1), and it reached 94.3% after five cycles [75]. Large channels constructed within {Cu-pz} MOFs decreased spatial site resistance, offering favorable sites for As^III^-capped Keggin arsenomolybdate accommodation [75]. In accordance with the intrinsic photocatalytic mechanism of POMs, photogenerated electrons migrated from the HOMO of oxygen to the LOMO of molybdenum and vanadium under UV irradiation. Concurrently, [As_2_^III^As^V^Mo_8_^VI^V_4_^IV^O_40_]^5−^ transitioned into its excited state, leading to the generation of electron–hole pairs, ^•^OH, and O_2_^•−^ for MB degradation.

An organic–inorganic hybrid material based on Mo_8_O_26_ (Cu_2_(L_1_)_2_(Mo_8_O_26_)_0.5_; L_1_:5-(pyrimidyl)tetrazolate) was synthesized via a hydrothermal reaction [76]. It achieved a significant 97.9% removal of MB (3.2 mg/L) within 7 min with 105 mg/L of Cu_2_(L_1_)_2_(Mo_8_O_26_)_0.5_, 200 mg/L of H_2_O_2_ (Table 1) [76]. After four cycles, there was only about a 1.9% decrease in removal efficiency (Table 1). Beyond the excitation of Cu_2_(L_1_)_2_(Mo_8_O_26_)_0.5_ that generated hole–electron pairs for active species formation (Equations (5)–(9)), MB also possessed the capability to reach an excited state (Equation (10)) and participated in a reaction with O_2_ to produce ^1^O_2_ (Equation (11)). Consequently, the subsequent decomposition of MB occurred under the influence of these active species (Equation (12)) [77]. With the same synthesis method as that of Cu_2_(L_1_)_2_(Mo_8_O_26_)_0.5_, 3D Cu^II^ hybrid materials based on Mo_8_O_26_^4−^, termed Cu_2_(L_2_)_3_(Mo_4_O_13_)_2_ (with L_2_ indicating 2,6-(1,2,4-triazole-4-yl)pyridine), removed 99.1% of the MB (3.2 mg/L) in 90 min under UV irradiation (Table 1) [78]. And the performance remained stable without significant decreases after five cycles. This UV-induced irradiation initiated a charge transfer within the N-donor ligand, leading to the generation of ^•^OH and O_2_^•−^ through interactions involving POM/organic ligands [79]. Ce_2_(BINDI)(Mo_6_O_19_) (H_4_BINDI: N,N′-bis(5-isophthalic acid)-1,4,5,8 -naphthalenediimide), obtained by heating reflux synthesis, removed 96.0% of MB (3.2 mg/L) after a 27 min visible irradiation period and the removal efficiency remained unchanged after 3 cycles (Table 1) [80]. Mo_6_O_19_^2−^ existed within the e^−^-deficient NDIs plane, acting as a reservoir for electrons and facilitating charge transfer. Additionally, core–shell-shell Fe_3_O_4_/Ag/POMs, composed of Fe_3_O_4_/Ag/[Cu(PCA)_2_(H_2_O)]H_2_[Cu(PCA)_2_(P_2_Mo_5_O_23_)]·4H_2_O (abbreviated as Fe_3_O_4_/Ag/Cu_2_(PCA)_4_(P_2_Mo_5_), PCA: pyridine-2-carboxamide), which was synthesized via the sonochemical method, removed 98.7% of the MB within 26 min under visible irradiation (Table 1) [81]. It decreased only by about 1.2% after six repeated tests. Ag functioned as a photogenerated electron acceptor prompted the interaction between chemisorbed molecular oxygen and photogenerated electrons, consequently yielding the creation of O_2_^•−^ [82]. This interaction notably contributed to the effective capture of photo-generated electrons.

Cu_2_(L_1_)_2_(Mo_8_O_26_)_0.5_ + UV → Cu_2_(L_1_)_2_(Mo_8_O_26_)_0.5_ (e^−^ + h^+^) (5)

Cu_2_(L_1_)_2_(Mo_8_O_26_)_0.5_ (e^−^ + h^+^) + H_2_O → Cu_2_(L_1_)_2_(Mo_8_O_26_)_0.5_ (e^−^) + ^•^OH + H^+^ (6)

Cu_2_(L_1_)_2_(Mo_8_O_26_)_0.5_ (e^−^) + H_2_O_2_ + H^+^ → Cu_2_(L_1_)_2_(Mo_8_O_26_)_0.5_ + ^•^OH + H_2_O (7)

Cu_2_(L_1_)_2_(Mo_8_O_26_)_0.5_ (e^−^) + O_2_ → Cu_2_(L_1_)_2_(Mo_8_O_26_)_0.5_ + O_2_^•−^ (8)

Cu_2_(L_1_)_2_(Mo_8_O_26_)_0.5_ (e^−^) + O_2_ + H^+^ → Cu_2_(L_1_)_2_(Mo_8_O_26_)_0.5_ + ^•^O_2_H (9)

MB + UV → MB*(10)

MB* + O_2_ → MB + ^1^O_2_ (11)

MB + ^1^O_2_, ^•^OH, ^•^O_2_H/O_2_^•−^ → degradation intermediates → CO_2_ + H_2_O (12)

Covalent organic frameworks (COFs) present another avenue for incorporating POMs into the framework to facilitate the photocatalytic activity of POMs. P@Ni-AndCOF and P@Cu-AndCOF were obtained through solvothermal synthesis using 4-functionalized porphyrins and Anderson-POM ([N(Bu)_4_]_4_[α-Mo_8_O_26_]), which eliminated 79.3% (P@Ni-AndCOF) and 89.0% (P@Cu-AndCOF) of the MB over a 660 min UV-Vis light irradiation period (Table 1) [83]. The heightened photocurrent response of P@Ni-AndCOF potentially accounted for its superior activity [84]. The pronounced electron-attracting ability of the Anderson-POM facilitated the transfer of excited electrons within P@M-AndCOF towards the Anderson-POM component, where the Anderson-POM fulfilled a pivotal role in electron storage during the photocatalysis process [37]. Subsequently, photogenerated electrons engaged with O_2_ to generate O_2_^•−^, while h^+^ interacted with H_2_O to give rise to ^•^OH, thereby facilitating the effective decomposition of MB.

**Figure 2:**
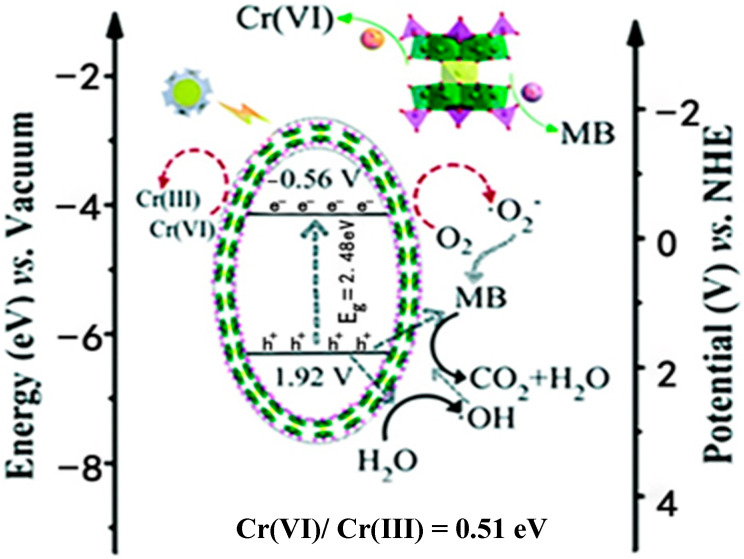
The possible mechanism of the simultaneous MB oxidation and Cr(VI) reduction with (Hbpp)_2_CoCd(P_4_Mo_6_)_2_ upon visible light irradiation. Adapted with permission from Ref. [85].

Among the extensive array of POMs, a distinct subset is represented by the reduced forms, known as heteropoly blues, which are characterized by their heightened electron density and broader spectral absorption, encompassing both the visible and near-infrared regions [85]. An particular example is the “hourglass” species [M(P_4_Mo_6_O_31_)_2_] (abbreviated as [M(P_4_Mo_6_)]), which is formed by linking two identical [P_4_Mo_6_O_31_]_12_^−^ units through bridging metal centers (M), thus featuring all reduced Mo atoms in a valence state of (+5) [86]. Based on this premise, a multifunctional photocatalyst of the “hourglass-type” POM ((H_2_bpp)_2_{[Na_4_(H_2_O)_5_][Co_0_._8_Cd_0_._2_(H_2_O)_2_][Cd[Mo_6_O_12_(OH)_3_(H_2_PO_4_)(HPO_4_)(PO_4_)_2_]_2_]}·2H_2_O, abbreviated as (Hbpp)_2_CoCd(P_4_Mo_6_)_2_) was obtained through a hydrothermal reaction for MB oxidation and Cr^6+^ reduction using visible light irradiation [85]. It achieved a 96.0% removal of MB (32 mg/L) and a 74.0% reduction in Cr^6+^ with 2000 mg/L catalyst over 180 min (Table 1), and the removal efficiency remained unchanged after five photocatalytic cycles. (Hbpp)_2_CoCd(P_4_Mo_6_)_2_ was irradiated to generate electrons and holes, which subsequently migrated to its surface and participated in redox reactions (Figure 2). Photogenerated electrons reduced Cr^6+^ to Cr^3+^, while photogenerated holes initiated the oxidation of MB. Notably, alongside h^+^, both ^•^OH and O_2_^•−^ contributed to the oxidation of MB. The concerted reduction in Cr^6+^ and oxidation of MB in this system effectively decreased the recombination of photogenerated electron–hole pairs, thereby fostering the separation of photogenerated charge carriers and optimizing the utilization of active species [87]. In a subsequent study by Huili Guo et al. in 2022 [88], two distinctive 3D Fe^2+^ frameworks, namely {H(4,4′bipy)_2_[Fe_4_(PO_4_)(H_2_O)_4_Na_6_][Fe_6_(H_2_O)_4_][(Mo_6_O_12_)(HPO_4_)_3_(PO_4_) (OH)_3_]_4_·5H_2_O} (Fe_4_Fe_6_(P_4_Mo_6_)_2_) and {H_3_(C_12_H_14_N_2_)_4_[Fe_4_(PO_4_)(H_2_O)_4_Na_4_][Fe_2_(Mo_6_O_12_ (HPO_4_)_3_(PO_4_)(OH)_3_)_4_]·6H_2_O} (Fe_4_Fe_2_(P_4_Mo_6_)_2_), were synthesized through solvothermal synthesis using P_4_Mo_6_ units as the foundations. They achieved outstanding removal of 98.0% ((Fe_4_Fe_6_(P_4_Mo_6_)_2_) and 99.0% (Fe_4_Fe_2_(P_4_Mo_6_)_2_) for MB (19 mg/L), and 95.6% ((Fe_4_Fe_6_(P_4_Mo_6_)_2_) and 82.0% (Fe_4_Fe_2_(P_4_Mo_6_)_2_) for Cr^6+^ (57 mg/L) within 180 min using 1000 mg/L catalyst and 555 mg/L of H_2_O_2_ (Table 1). In addition, Fe_4_Fe_6_(P_4_Mo_6_)_2_) and (Fe_4_Fe_2_(P_4_Mo_6_)_2_ also demonstrated high recyclability with little decrease after ten cycles.

**Table 1:** Various procedures for photocatalytic degradation of MB with POM-based catalyst.

Catalyst	Synthesis Method	Irradiation	Catalyst Dosage (mg/L)	MB Dosage (mg/L)	pH	Time (min)	Removal Efficiency (%)		Ref.
							1st	nth	
PMo_12_V_3_	Hydrothermal	UV	500	10	-	65	99.3	~/5th	[53]
PMo_11_V/g-C_3_N_4_	Dipping	Vis	400	10	-	120	94.7	-	[56]
P_2_W_18_Sn_3_/Nd-TiO_2_	One-Pot Synthesis	UV	-	10	3	5	91.0	-	[57]
Mn-BTC@Ag_5_[BW_12_]	Grinding	UV	500	15	-	140	95.6	~/5th	[62]
Ag-BTC@Ag_5_[BW_12_]	Grinding	UV	500	15	-	140	94.4	~/5th	[66]
Zn-BTC@Ag_5_[BW_12_]	Grinding	UV	500	15	-	140	96.1	~/5th	[67]
[Ag_5_(pz)_6_][BW_12_]	Hydrothermal	UV	500	15	-	140	93.2	~/5th	[68]
[Hpip]_2_._5_[BW_12_]	Heating reflux	Vis	800	20	-	24	97.1	90.0/5th	[74]
[Cu_3_(pz)_4_]_2_[As_3_Mo_8_V_4_]_2_	Hydrothermal	UV	400	10	-	120	97.8	94.3/5th	[75]
Cu_2_(L_1_)_2_(Mo_8_O_26_)_0_._5_	Hydrothermal	UV	105	3.2	6.8	7	97.9	96.0/4th	[76]
Cu_2_(L_2_)_3_(Mo_4_O_13_)_2_	Hydrothermal	UV	500	3.2	-	90	99.1	~/5th	[78]
Ce_2_(BINDI)(Mo_6_O_19_)	Heating reflux	Vis	300	3.2	-	27	96.0	~/3rd	[80]
Fe_3_O_4_/Ag/Cu_2_(PCA)_4_(P_2_Mo_5_)	Sonochemical	Vis	166.7	15	-	26	98.7	97.5/6th	[81]
P@Cu-AndCOF P@Ni-AndCOF	Solvothermal	UV-Vis	100	50	-	660	89.0 79.3	-	[83]
(Hbpp)_2_CoCd(P_4_Mo_6_)_2_	Hydrothermal	Vis	2000	32	6.8	180	96.0	~/5th	[85]
Fe_4_Fe_6_(P_4_Mo_6_)_2_ Fe_4_Fe_2_(P_4_Mo_6_)_2_	Solvothermal	Vis	1000	19	-	180	98.0 99.0	~/10th	[88]

UV: Ultraviolet irradiation; Vis: visible light irradiation; UV-Vis: ultraviolet- visible light irradiation. 1st: Dye removal efficiency with the catalyst’s first use for degradation; nth: dye removal efficiency after the catalyst has been recycled for n times; ~: dye removal efficiency was almost unchanged after n cycles of catalyst.

### 2.2. Rhodamine B (RhB)

**Figure 3:**
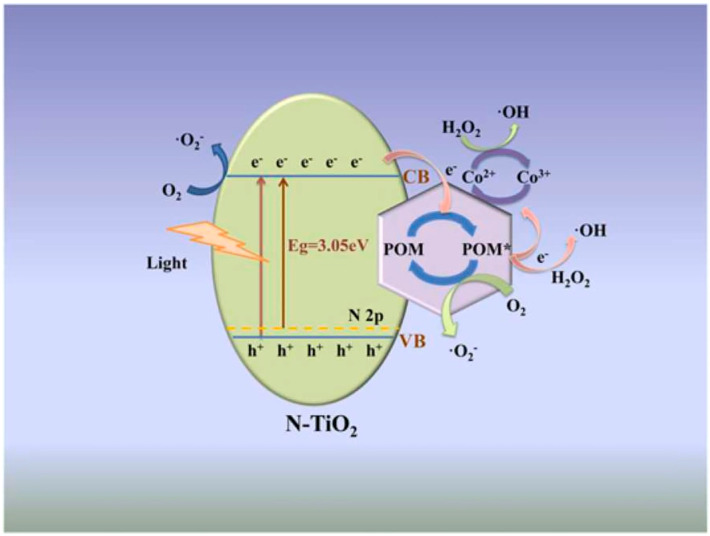
The mechanism diagram of RhB degradation by Co-PMo_12_/N-TiO_2_ (POM * meant the reduced state POM). Adapted with permission from Ref. [98].

Frequently employed as a photocatalyst, TiO_2_ possesses a relatively wide bandgap ranging from 3.0 to 3.2 eV [89,90,91]. Nevertheless, its photocatalytic efficiency can be significantly enhanced through the combination of POMs, which offer customizable electronic characteristics capable of tuning TiO_2_’s bandgap [92,93,94]. This strategic modulation amplifies the overall photocatalytic performance. K_5_CoW_12_/TiO_2_ (CoW_12_/TiO_2_, 5000 mg/L) synthesized by sol-gel/hydrothermal method achieved the complete removal of RhB (15 mg/L) within 30 min at pH = 5 under visible light irradiation (Table 2) [95]. And the performance remained unchanged after four cycles. Within this system, O_2_^•−^ and photogenerated holes emerged as the primary active species driving RhB degradation [96,97]. Similarly, effective RhB removal under visible light conditions was achieved with a remarkable 98.0% removal within 40 min by employing the Fenton-like heterogeneous catalyst Co-H_3_PMo_12_O_40_/N-TiO_2_ (Co-PMo_12_/N-TiO_2_) (400 mg/L) in conjunction with the addition of 1110 mg/L of H_2_O_2_ (Table 2) [98], and Co-PMo_12_/N-TiO_2_, obtained through one-pot synthesis, was of great stability (Table 2). Beyond facilitating photoinduced valence electron transitions, POMs also functioned as electron acceptors, effectively curtailing the recombination of photo-generated holes and electrons (Figure 3) [98]. Upon excitation by visible light, valence electrons within N-TiO_2_ migrated to its CB (Equation (13)). These electrons subsequently underwent rapid transfer to Co-POM, causing it to transition into a reduced state (POM*) (Equation (14)) due to the robust hydrogen bonding between Co-PMo_12_ and N-TiO_2_ [77]. This phenomenon substantially suppressed the recombination of photo-generated electrons and holes, thereby facilitating the progression of reactions. During this process, photo-generated electrons reacted with oxygen, leading to the formation of O_2_^•−^ (Equation (15)). Concurrently, POM* in its reduced state reacted with O_2_, yielding O_2_^•−^ and reverting POM* to POM (Equation (16)), thus entering the Co^2+^/Co^3+^ cycle (Equations (17) and (18)) [99]. The catalytic effect of Co^2+^ results in the generation of Co^3+^ and ^•^OH from H_2_O_2_ (Equation (18)). This collaborative interplay between ^•^OH, holes, and O_2_^•−^ culminates in the degradation of RhB (Figure 3).

Co-PMo_12_/N-TiO_2_ + h*v* → e^−^ + h^+^ (13)

PMo_12_ + e^−^ → PMo_12_* (14)

O_2_ + e^−^ → O_2_^•−^ (15)

PMo_12_* + O_2_ → PMo_12_ + O_2_^•−^ (16)

Co^3+^ + PMo_12_* → Co^2+^ + PMo_12_ (17)

Co^2+^ + H_2_O_2_ → Co^3+^ +^•^OH + OH^−^ (18)

**Figure 4:**
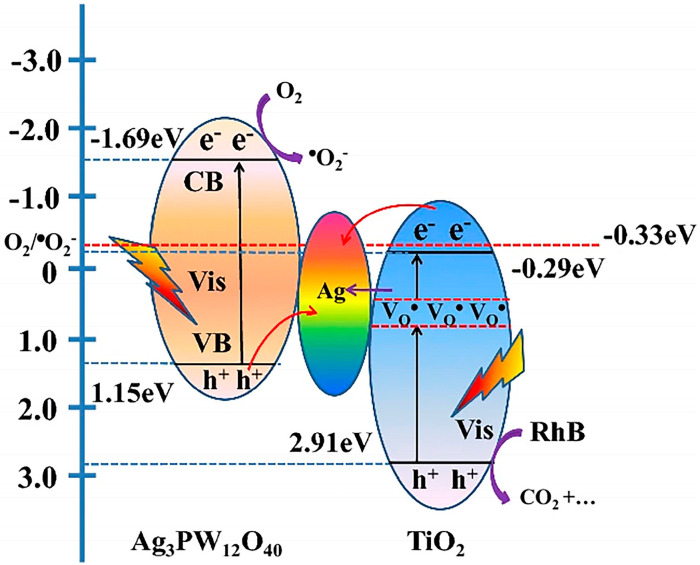
The photocatalytic degradation mechanism of RhB with Z-system photocatalyst Ag_3_PW_12_/TiO_2_. Adapted with permission from Ref. [101].

**Figure 5:**
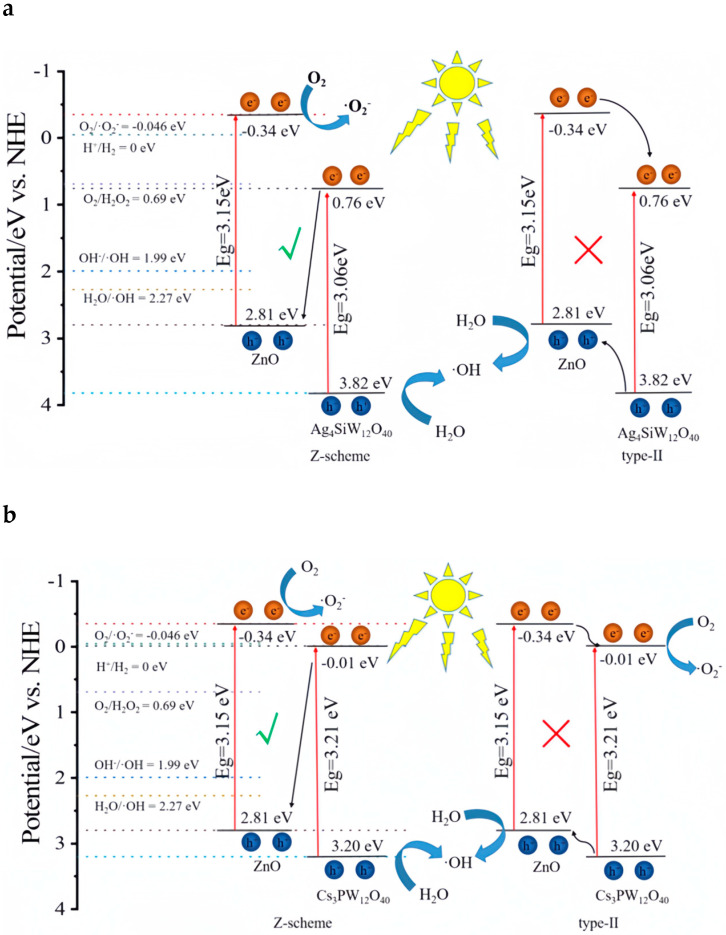
Possible photocatalytic mechanism of (**a**) ZnO/Ag_4_SiW_12_ and (**b**) ZnO/Cs_3_PW_12_ under simulated sunlight irradiation. Adapted with permission from Ref. [106].

In addition to their photo-responsive attributes, POMs possess the capability to engage with other materials, facilitating the creation of interfaces that enable the manipulation of both electronic structure and the chemical environment at the interface [100]. Shengnan Cai et al. [101] employed Ag_3_PW_12_O_40_ (Ag_3_PW) in tandem with TiO_2_ through hydrothermal synthesis method to craft the Z-scheme Ag_3_PW_12_O_40_/TiO_2_ (Ag_3_PW_12_/TiO_2_) system. This configuration aimed to bolster charge carrier separation and amplify visible-light catalytic activity [101]. Ag_3_PW_12_/TiO_2_ achieved 95.0% removal of RhB (10 mg/L) within 120 min with Ag_3_PW_12_/TiO_2_ (1000 mg/L) under visible irradiation, and the removal efficiency was almost unchanged after four cycles (Table 2). The degradation was primarily governed by h^+^, accompanied by a minor contribution from O_2_^•−^ (Figure 4). Under visible irradiation, photogenerated electrons migrated from CB of TiO_2_ to that of the metallic Ag due to the more negative CB potential of TiO_2_ in comparison to Ag’s Fermi level (Figure 4). Concurrently, holes within the VB of Ag_3_PW_12_ transferred to Ag^+^, engaging in binding with electrons due to Ag’s more positively positioned Fermi level [102]. Within the configuration of the Z-scheme Ag_3_PW_12_/TiO_2_, Ag played the pivotal role of a bridging agent [103,104,105]. This role effectively facilitated charge transfer through Ag_3_PW_12_, thereby promoting the separation of electrons and holes. Expanding beyond the realm of TiO_2_, the band structure alignment of Ag_4_SiW_12_O_40_ (Ag_4_SiW_12_) and Cs_3_PW_12_O_40_ (Cs_3_PW_12_) with ZnO is noteworthy [106]. This alignment enabled the creation of ZnO/Ag_4_SiW_12_ and ZnO/Cs_3_PW_12_ systems, which were synthesized through the precipitation method and achieved 92.3% (ZnO/Ag_4_SiW) and 72.7% (ZnO/Cs_3_PW_12_) of RhB removal within 60 min under simulated sunlight irradiation, whereas the performance of ZnO alone yielded 17.2% removal of RhB (Table 2) [106]. After recycling three times, the removal efficiency decreased to 84.8% (ZnO/Ag_4_SiW) and 65.3% (ZnO/Cs_3_PW_12_), respectively. The CB potential of ZnO was more negative than that of O_2_/O_2_^•−^, leading to the reaction of O_2_ with electrons on ZnO’s CB, generating O_2_^•−^ [107,108]. Furthermore, VB potential surpassed that of OH^−^/^•^OH and H_2_O/^•^OH, thereby fostering the formation of ^•^OH on the VB of Cs_3_PW_12_/AgPW. In this scenario, electrons on the CB of Cs_3_PW_12_/AgPW were directed to the CB of ZnO (Figure 5a,b).

POMOFs, anchored on SiW_12_O_40_^4−^ (SiW_12_), exhibit a distinctive potential for the photocatalytic degradation of dyes due to the pronounced negative charges and the reversible multi-electron transfer characteristics inherent to SiW_12_ [109]. Co_2_(3,3′-bpy)(3,5′-bpp)(4,3′-bpy)_3_[SiW_12_O_40_] ([Co_2_(bpy)_3_][SiW_12_]); 4,3′-bpy (4,3′-dipyridine); 3,5′-bpp (3,5′-bis(pyrid-4-yl)pyridine); and 3,3′-bpy (3,3′-bis(pyrid-4-yl) dipyridine)), synthesized by the hydrothermal method, achieved a 92.0% removal of RhB (50 mg/L) at 120 min under UV irradiation (Table 2) [109]. The removal efficiency did not decrease significantly after three cycles (Table 2). When irradiated, the SiW_12_ within [Co_2_(bpy)_3_][SiW_12_] was prompted into an excited state, thereby giving rise to the generation of holes and electrons through charge transfer from O to W in W-O-W ligands. These active species were pivotal for the ensuing RhB degradation. In addition, the hybrid complex, [Cu_8_Cl_5_(CPT)_8_ (H_2_O)_4_](HSiW_12_)(H_2_O)_20_(CH_3_CN)_4_}_n_ (abbreviated as [Cu_8_(CPT)_8_](SiW_12_)(CH_3_CN)_4_; HCPT: 4-(4-carboxyphenyl)-1,2,4-triazole) accomplished 96.6% of RhB removal (10 mg/L) within 70 min by utilizing 3.4 × 10^4^ mg/L of H_2_O_2_ under visible irradiation (Table 2) [110]. In contrast, the efficiency was only 46.4% with [Cu_8_(CPT)_8_](SiW_12_)(CH_3_CN)_4_ alone. [Cu_8_Cl_5_(CPT)_8_ (H_2_O)_4_], synthesized by the solvothermal method, maintained its original structure in comparison to the initial state. Moreover, the activity of [Cu_8_Cl_5_(CPT)_8_ (H_2_O)_4_] exhibited a negligible decrease after undergoing four cycles (Table 2). The introduction of H_2_O_2_, which is capable of accepting electrons, played a pivotal role in enhancing the degradation [111]. This was attributable to its capture of photogenerated electrons and the reduction in hole–electron pair recombination [112]. However, excessive H_2_O_2_ content can potentially quench the generated ^•^OH [113]. 

In parallel with SiW_12_, the POMOFs rooted in BW_12_ demonstrated comparable potential for the photocatalytic degradation of RhB. Specifically, core–shell configurations, such as Ag-BTC@Ag_5_[BW_12_] [66], Zn-BTC@Ag_5_[BW_12_] [67], and Mn-BTC@Ag_5_[BW_12_] [62], displayed noteworthy removal efficiencies of 90.6%, 91.1%, and 94.1%, respectively, for RhB (15 mg/L) within 140 min using 500 mg/L of catalyst under UV irradiation (Table 2). Additionally, [Cu(en)_2_(H_2_O)][{Cu(pdc)(en)}(Cu(en)_2_)(BW_12_)]·2H_2_O (abbreviated as [Cu_3_(en)]_4_(pdc)[BW_12_]) and [{Cu^I^_5_(pz)_6_(H_2_O)_4_}(BW_12_)] (abbreviated as [Cu^I^_5_(pz)_6_][BW_12_]), prepared by the hydrothermal method, achieved RhB removal efficiencies of 91.3% and 92.6%, respectively, when subjected to 150 min of UV light irradiation with 500 mg/L catalyst and 10 mg/L RhB (Table 2) [114]. Though the synthesized methods were different, Ag-BTC@Ag_5_[BW_12_] [66], Zn-BTC@Ag_5_[BW_12_] [67], Mn-BTC@Ag_5_[BW_12_] [62] [Cu_3_(en)]_4_(pdc)[BW_12_]) and [Cu^I^_5_(pz)_6_][BW_12_] were of great stability (Table 2). Notably, [Cu_3_(pz)_4_]_2_[As_3_Mo_8_V_4_]_2_ accomplished a 97.4% RhB removal (10 mg/L) in 120 min using 400 mg/L of catalyst under UV irradiation (Table 2) [75]. And the removal efficiency of RhB was 92.7% after five cycles, with only a slight decrease of 4.7%.

**Figure 6:**
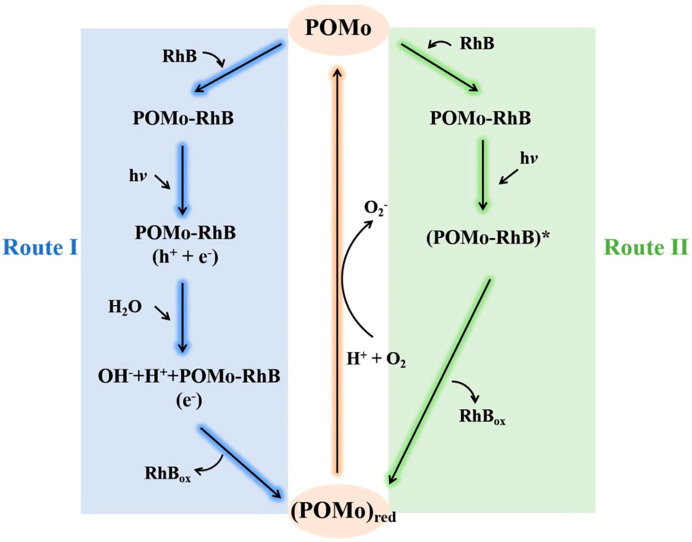
The degradation path of RhB (* meant excited state). Adapted with permission from Ref. [115].

{P_2_Mo_5_}-based POMOFs, exemplified by [H4′4-bipy)_2_][H_2_P_2_Mo_5_O_23_] [Cu(4′4-bipy)_2_]·18H_2_O (abbreviated as Cu(bipy)_4_(P_2_Mo_5_)), achieved 89.6% removal of RhB (30 mg/L) within 180 min through visible light irradiation (Table 2) [115]. Cu(bipy)_4_(P_2_Mo_5_), obtained through the hydrothermal reaction, exhibited excellent stability, and there was no significant decline in catalytic activity observed even after four cycles. Evidently, the presence of POMs-RhB complexes prior to degradation facilitated the interaction of active species with RhB during the photocatalytic process [116]. Intriguingly, aside from the active species generated, RhB can also undergo degradation through the excited POMs (Figure 6) [115]. Additionally, Cu_2_(L_2_)_3_(Mo_4_O_13_)_2_ removed 98.7% of RhB (4.79 mg/L) within 90 min under UV irradiation (Table 2) [78]. Moreover, P@Ni-AndCOF showcased a 71.3% removal of RhB (100 mg/L) within 660 min under UV-Vis light irradiation (Table 2), while P@Cu-AndCOF yielded an 83.0% removal of RhB (Table 2) [83].

**Table 2:** Various procedures for photocatalytic degradation of RhB with POM-based catalyst.

Catalyst	Synthesis Method	Irradiation	Catalyst Dosage (mg/L)	RhB Dosage (mg/L)	pH	Time (min)	Removal Efficiency (%)		Ref.
							1st	nth	
CoW_12_/TiO_2_	Sol-gel/hydrothermal	Vis	5000	15	5	30	100	~/4th	[95]
Co-PMo_12_/N-TiO_2_	One-pot synthesis	Vis	400	10	7	40	98.0	~/4th	[98]
Ag_3_PW_12_/TiO_2_	Hydrothermal	Vis	1000	10	-	120	95.0	~/4th	[101]
ZnO/Ag_4_SiW ZnO/Cs_3_PW_12_	Precipitation	Simulated sunlight	300	50	-	60	92.3 72.7	84.8/3rd 65.3/3rd	[106]
Co_2_(bpy)_3_][SiW_12_]	Hydrothermal	UV	400	50	-	120	92.0	~/3rd	[109]
[Cu_8_(CPT)_8_](SiW_12_)(CH_3_CN)_4_	Solvothermal	Vis	375	10	-	70	96.6	~/4th	[110]
Ag-BTC@Ag_5_[BW_12_]	Grinding	UV	500	15	-	140	90.6	~/5th	[66]
Zn-BTC@Ag_5_[BW_12_]	Grinding	UV	500	15	-	140	91.1	~/5th	[67]
Mn-BTC@Ag_5_[BW_12_]	Grinding	UV	500	15	-	140	94.1	~/5th	[62]
[Cu_3_(en)]_4_(pdc)[BW_12_] [Cu^I^_5_(pz)_6_][BW_12_]	Hydrothermal	UV	500	10	-	150	91.3 92.6	~/5th	[114]
[Cu_3_(pz)_4_]_2_[As_3_Mo_8_V_4_]_2_	Hydrothermal	UV	400	10	-	120	97.4	92.7/5th	[75]
Cu(bipy)_4_(P_2_Mo_5_)	Hydrothermal	Vis	300	30	-	180	89.6	~/4th	[115]
Cu_2_(L_2_)_3_(Mo_4_O_13_)_2_	Hydrothermal	UV	500	4.79	-	90	98.7	~/4th	[78]
P@Ni-AndCOF P@Cu-AndCOF	Solvothermal	UV-Vis	100	50	-	660	71.3 83.0	-	[83]

Vis: Visible light irradiation; UV: ultraviolet irradiation; UV-Vis: ultraviolet- visible light irradiation. 1st: Dye removal efficiency with the catalyst’s first use for degradation; nth: dye removal efficiency after the catalyst has been recycled for n times; ~: dye removal efficiency was almost unchanged after n cycles of catalyst.

### 2.3. Methyl Orange (MO)

**Figure 7:**
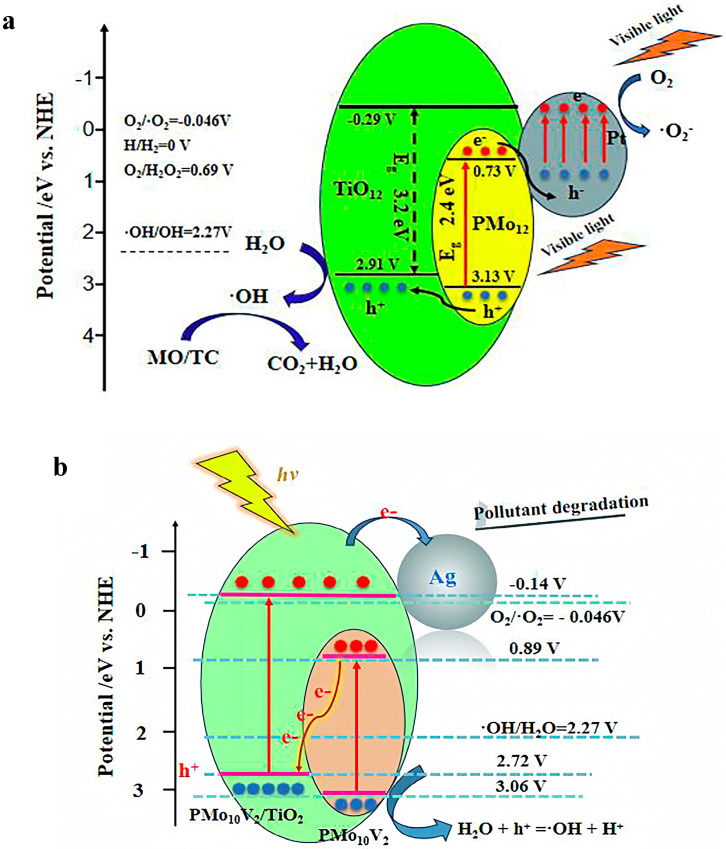
The possible photocatalytic degradation mechanism of MO with (**a**) Pt/PMo_12_/TiO_2_ [119] and (**b**) Ag/PMo_10_V_2_/TiO_2_ [124]. Adapted with permissions from Refs. [119,124].

Keggin-type POMs, particularly H_3_PMo_12_O_40_ (PMo_12_), characterized by a narrow energy gap (Eg) of 2.4 eV, are well known for its exceptional stability and remarkable visible-light absorption attributes. Pt/PMo_12_/TiO_2_ composite nanofibers were fabricated via the electrospinning/calcination route and photoreduction for MO degradation [117,118,119]. Remarkably, it achieved an 88.1% removal of MO within 180 min at pH 1, employing 1000 mg/L of Pt/PMo_12_/TiO_2_ and 20 mg/L of MO (Table 3) [119]. The PMo_12_’s VB potential (3.13 eV) was notably more positive than that of TiO_2_ (2.91 eV), facilitating the migration of holes generated by PMo_12_ into TiO_2_’s domain (Figure 7a). In contrast, the photogenerated electrons did not react with O_2_ due to PMo_12_’s higher LUMO value (0.73 V vs. NHE) in comparison to O_2_/O_2_^•−^ (−0.046 V vs. NHE). Consequently, photogenerated electrons produced by PMo_12_ combined with the plasmonic holes within Pt [120,121,122]. Subsequently, the plasma electrons within Pt were directly captured by absorbed O_2_ on the surface, resulting in the formation of O_2_^•−^. MO was degraded through the collaboration of ^•^OH and O_2_^•−^. Noble metal deposition with Bi further augmented the photocatalytic performance. Bi/PMo_12_-doped TiO_2_ (Bi/PMo_12_/TiO_2_, denoted as x = 10, 20, and 30), obtained through the electrospinning/calcination and hydrothermal methods, removed 92.5% of MO within 180 min at pH 1 under visible light irradiation, utilizing 1000 mg/L Bi/PMo_12_/TiO_2_ and 40 mg/L MO (Table 3) [123]. In comparison, single TiO_2_, PMo_12_-doped TiO_2_ (PMo_12_/TiO_2_), and Bi/TiO_2_ achieved removal rates of 14.6%, 18.2%, and 64.6%, respectively [123]. And both Pt/PMo_12_/TiO_2_ [119] and Bi/PMo_12_/TiO_2_ [123] were of great stability, with little decreases in activity observed even after five cycles (Table 3). Complete MO removal was realized within 30 min employing 1000 mg/L of Ag/PMo_10_V_2_O_40_^5−^/TiO_2_ (Ag/PMo_10_V_2_/TiO_2_) and 20 mg/L of MO under visible light irradiation (Table 3) [124]. Ag/PMo_10_V_2_/TiO_2_ was synthesized by heating reflux and photoreduction [124]. Notably, an observed S-type heterojunction within Ag/PMo_10_V_2_/TiO_2_ facilitated the excitation of both PMo_10_V_2_ and PMo_10_V_2_/TiO_2_ by visible irradiation (Figure 7b). The photogenerated electrons from PMo_10_V_2_’s LUMO migrated to the VB of PMo_10_V_2_/TiO_2_, resulting in the subsequent combination with the holes present in the VB of PMo_10_V_2_/TiO_2_. Concurrently, photoelectrons generated on Ag/PMo_10_V_2_-TiO_2_ transported to the surface of Ag nanoparticles through Schottky junctions, engaging in interaction with O_2_, leading to the formation of O_2_^•−^ (Figure 7b). Remarkably, a 98.4% removal efficiency of MO was achieved within just 5 min with Sandwich-type POM/TiO_2_ (P_2_W_18_Sn_3_/Nd-TiO_2_) at pH = 3 under UV irradiation, and the removal efficiency was 95.0% after five cycles (Table 3) [57]. MO was anionic, whereas TiO_2_’s pH_pzc_ was 6.8, rendering it positively charged in acidic conditions (pH < 6.8). Thus, higher response rates were observed in acidic settings owing to the electrostatic attraction between TiO_2_ and MO [125]. The magnetic microsphere of Fe_3_O_4_@SiO_2_@[TiO_2_/H_3_PW_12_O_40_]_10_ (Fe_3_O_4_@SiO_2_@[TiO_2_/PW_12_]_10_) was synthesized at room temperature using a Layer-by-Layer method. It achieved a 83.9% removal of MO under UV irradiation at pH 2, utilizing 2000 mg/L Fe_3_O_4_@SiO_2_@[TiO_2_/PW_12_]_10_ and 10 mg/L MO (Table 3) [126]. In contrast, little degradation occurred with Fe_3_O_4_@SiO_2_ alone. The exceptional performance was attributed to the synergistic action of TiO_2_ and PW_12_. And Keggin-typed SiW_10_ exhibited characteristic absorption below 400 nm, and its absorption peak gradually broadened with increasing Co content [127]. Based on the remarkable UV-Vis absorption of Co_2_Co_4_(SiW_10_O_37_)_2_, [Co_2_Co_4_(SiW_10_O_37_)_2_/Fe_2_O_3_] (Co_2_Co_4_(SiW_10_)_2_/Fe_2_O_3_) achieved a 76.2% removal of MO under UV irradiation within 90 min at pH = 1 (Table 3) [128]. And the removal efficiency reached 69.4% after three cycles, exhibiting a decrease of 6.8%.

POMOFs originating from BW_12_, namely [Ag_5_(pz)_6_][BW_12_] [68], Ag-BTC@Ag_5_[BW_12_] [66] and Zn-BTC@Ag_5_[BW_12_] [67], demonstrated exceptional efficacy in the removal of MO at concentrations of 15 mg/L. When subjected to UV irradiation, they achieved removal efficiencies of 90.9%, 92.4%, and 95.2%, respectively, within 140 min, when utilizing a catalyst concentration of 500 mg/L and 15 mg/L of MB (Table 3). Exceptional stability was observed in these three catalysts, as the removal efficiency of MO remained unchanged even after undergoing five cycles (Table 3). Furthermore, [Cu_3_(pz)_4_]_2_[As_3_Mo_8_V_4_]_2_, built upon on [As_2_^III^As^V^Mo_8_^VI^V_4_^IV^O_40_]^5−^, demonstrated an impressive MO removal of 96.8% within 120 min under UV irradiation. And the removal efficiency remained at 91.4% after undergoing five cycles (Table 3) [75].

**Figure 8:**
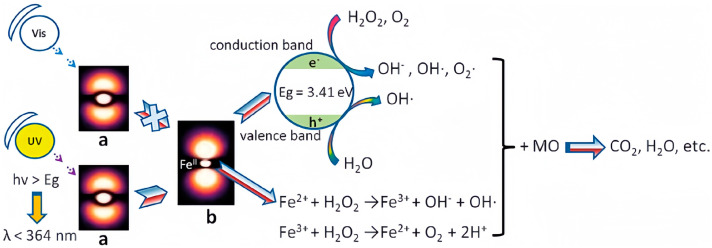
The possible photocatalytic degradation mechanism MO of with the heterogeneous Fenton-like catalyst of (DETA)_3.5_Fe(P_4_Mo_6_)_2_ (a meant (DETA)_3.5_Fe(P_4_Mo_6_)_2_; b meant excited state (DETA)_3.5_Fe(P_4_Mo_6_)_2_). Adapted with permission from Ref. [131].

POMs feature extensive electron-donating conjugation systems, enabling the stabilization of Fe^2+^ and thus enhancing the functionality of the Fenton system [129,130]. Exploiting this attribute, the Sandwich-type phosphomolybdate [H_4_MoV_6_O_15_(PO_4_)_4_]^8−^ was employed to create heterogeneous Fenton-like systems for MO removal [131]. The compound (H_3_O)_3.5_(H_3_DETA)_3.5_{Fe^II^[H_4_Mo^V^_6_O_15_(PO_4_)_4_]_2_} (abbreviated as (DETA)_3.5_Fe(P_4_Mo_6_)_2_, EDTA = diethylenetriamine), synthesized through hydrothermal reaction, exhibited remarkable performance by removal of 99.8% of the MO (20 mg/L) within 20 min with the addition of H_2_O_2_ (Table 3) [131]. In comparison, the removal efficiency was only 20.0% with H_2_O_2_ alone. The presence of metal–oxo clusters in POMs contributes to extensive electron donation, creating a positively charged environment around Fe^2+^ [129,130]. Consequently, the oxidation of Fe^2+^ to Fe^3+^ was significantly inhibited under both non-illuminated and visible light conditions [131]. Specifically, under UV irradiation, the charge transfer from O to Mo (i.e., Fe→O→Mo) reduced the electron density surrounding Fe^2+^, enabling Fe^2+^ to react with H_2_O_2_ to form Fenton reagents (Figure 8).

**Table 3:** Various procedures for photocatalytic degradation of MO with POM-based catalysts.

Catalyst	Synthesis Method	Irradiation	Catalyst Dosage (mg/L)	MO Dosage (mg/L)	pH	Time (min)	Removal Efficiency (%)		Ref.
							1st	nth	
Pt/PMo_12_/TiO_2_	Electrospinning/calcination and photoreduction	Vis	1000	20	1	180	88.1	~/5th	[119]
Bi/PMo_12_/TiO_2_	Electrospinning/calcination and hydrothermal	Vis	1000	40	1	180	92.5	~/8th	[123]
Ag/PMo_10_V_2_/TiO_2_	Heating reflux and photoreduction	Vis	1000	20	-	30	100	-	[124]
P_2_W_18_Sn_3_/Nd-TiO_2_	One-Pot Synthesis	UV	-	10	3	5	98.4	95.0/5th	[57]
Fe_3_O_4_@SiO_2_@[TiO_2_/PW_12_]_10_	Layer-by-Layer method	UV	2000	10	2	100	83.9	-	[126]
Co_2_Co_4_(SiW_10_)_2_/Fe_2_O_3_	Precipitation	UV	1000	10	1	90	76.2	69.4/3rd	[128]
[Ag_5_(pz)_6_][BW_12_]	Hydrothermal	UV	500	15	-	140	90.9	~/5th	[68]
Ag-BTC@Ag_5_[BW_12_]	Grinding	UV	500	15	-	140	92.4	~/5th	[66]
Zn-BTC@Ag_5_[BW_12_]	Grinding	UV	500	15	-	140	95.2	~/5th	[67]
[Cu_3_(pz)_4_]_2_[As_3_Mo_8_V_4_]_2_	Hydrothermal	UV	400	10	-	120	96.8	91.4/5th	[75]
(DETA)_3.5_Fe(P_4_Mo_6_)_2_	Hydrothermal	UV	60	20	-	20	99.8	-	[131]

Vis: Visible light irradiation; UV: ultraviolet irradiation; 1st: dye removal efficiency with the catalyst’s first use for degradation; nth: dye removal efficiency after the catalyst has been recycled for n times; ~: dye removal efficiency was almost unchanged after n cycles of catalyst.

## 3. Photodegradation Mechanism of Dyes and Enhancement Strategies for POM-Based Materials

### 3.1. Photodegradation Mechanism of Dyes

**Figure 9:**
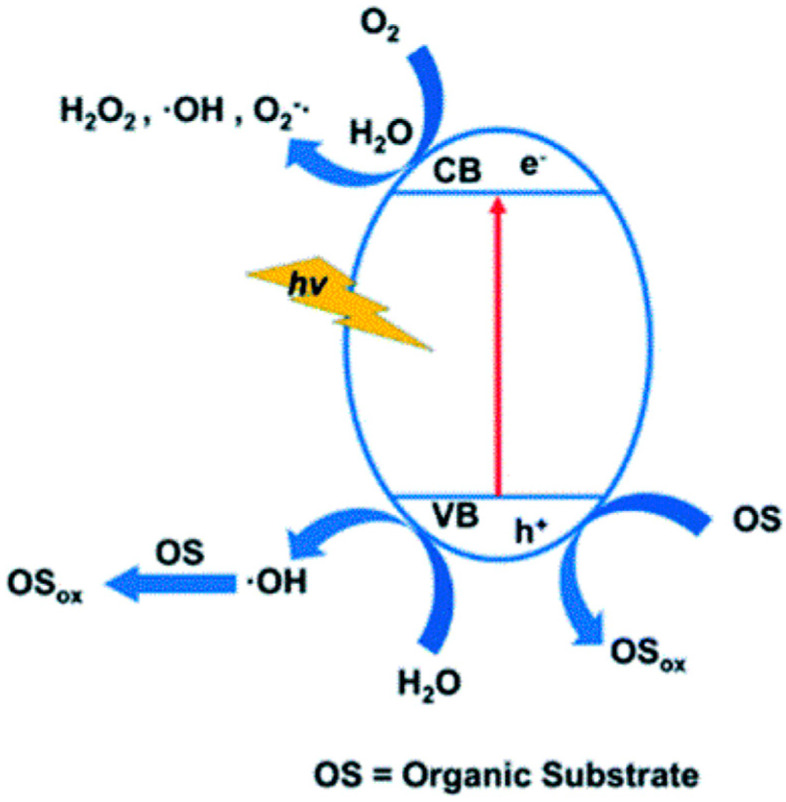
The photocatalytic mechanism of organic substrate with POMs. Adapted with permission from Ref. [39].

Metals within POMs predominantly exhibit a d0 electron configuration, leading to ligand-to-metal charge transfer upon exposure to light irradiation [40]. Moreover, for most POMs, a distinct energy gap emerges between the HOMO of oxygen and the LUMO of metals within metal–oxo clusters. As a result, the activation of most POMs necessitates ultraviolet or near-ultraviolet light irradiation [40]. For instance, in the case of PMo_12_V_3_, when photon energy matches or exceeds the bandgap, electrons transition from O^2−^ (2p) orbitals to Mo^6+^ (5d) orbitals, inducing PMo_12_V_3_ to enter an excited state and generate electron–hole pairs [53]. The photogenerated electrons in CB gain high energy and mobility, while photogenerated holes in VB possess potent oxidative capabilities [132,133,134]. If the VB potential of POMs exceeds that of H_2_O/^•^OH (2.7 V vs. NHE), these photogenerated holes would react with water, producing ^•^OH [106,135]. Alternatively, photogenerated electrons engage in reduction reactions, converting O_2_ into O_2_^•−^, facilitated by the more negative potential of CB of POMs compared to that of O_2_/^•^O_2_^−^ (−0.046 V vs. NHE) [55,107]. Subsequently, O_2_^•−^ react with water to generate ^•^OH (Figure 9) [39,136]. These dynamic active species initiate the attack and breakdown of dyes, efficiently converting them into smaller, less harmful byproducts, such as carbon dioxide and water [31]. Alongside the generated active species, excited POMs can also contribute to the degradation of dyes [137,138].

The combination of POMs with specific materials presents an alternative for enhancing the light absorption capabilities and photocatalytic activity of POM-based catalysts. For example, the coupling of Anderson-POM ([N(Bu)_4_]_4_[α-Mo_8_O_26_]) with a visible light-absorbing porphyrin leads to the creation of the photoreactive COF catalyst (P@Ni-AndCOF), which demonstrates visible light catalysis activity [83]. Additionally, the energy band structures, interface characteristics, and charge migration properties can also be modified by integrating POMs with different materials. Notably, the fabrication of a Z-scheme Ag_3_PW_12_/TiO_2_ (Figure 4) and the establishment of an S-type heterojunction in Ag/PMo_10_V_2_/TiO_2_ (Figure 7b) [101,124]. Upon light excitation, both POMs and carriers generate electron–hole pairs [39,139]. The electrons originating from the CB of POMs migrate to VB of the partnering carriers and combine with its holes. Concurrently, holes in VB of POMs remain localized within the POMs structure, participating in oxidation processes or promoting the generation of ^•^OH. Alternatively, photogenerated electrons of the partnering carriers combine with POMs’ holes. Then, the photogenerated electrons on CB engage in the formation of O_2_^•−^ [140].

Despite the removal of dyes in photocatalytic systems, the degradation pathways, and the specific roles of reactive oxygen species (ROS), such as h^+^, ^•^OH and O_2_^•−^, in their degradation are challenging to ascertain. These dyes have complex organic structures, making it difficult to analyze their degradation pathways as chemical bonds and interactions among functional groups can lead to various reactions. Additionally, degradation pathways are influenced by environmental conditions (such as temperature, pH, etc.), degradation methods, and catalysts. Variations in these factors can result in different degradation pathways, increasing the complexity of the research. Consequently, different researchers may propose different degradation mechanisms. For example, in the case of the degradation of Rhodamine B, Pengxiang Lei et al. [141] suggested that within the POM(Na_3_PW_12_O_40_)-resin/H_2_O_2_/visible light system, it underwent degradation through intermediates like N-ethyl-N′-ethyl-rhodamine, N, N-diethyl-N′-ethylrhodamine, and N, N-diethyl-rhodamine. While Zhentao Yu et al. [142] proposed a stepwise degradation mechanism involving the gradual loss of C_2_H_5_ units, facilitated by intermediates such as N, N′,N′-triethylrhodamine; N, N′-diethylrhodamine; and N-ethylrhodamine. The lack of consistent results not only complicates the determination of the exact degradation pathways but also hinders the understanding of the precise roles of ROS and POMs in the degradation process.

POMs, despite their diverse structures, typically possess small specific surface areas (1–10 m^2^·g^−1^) [33]. Consequently, specific surface area seems to have a minimal impact on their adsorption performance. For example, in the case of Ag_5_BW_12_ and PMo_11_V, under dark conditions, their removal efficiencies for MB were 3.8% (Ag_5_BW_12_) [62] and 8.0% (PMo_11_V) [56], respectively, with minimal variation observed. Nevertheless, when examining various POMs types, significant disparities in photocatalytic activity emerge. Notably, under visible light irradiation, Keggin-type Ag_5_[BW_12_O_40_] degraded 39.3% of the RhB within 140 min [62], while Dawson-type KNa-P_8_W_48_ accomplished a 91.0% removal of the RhB within 180 min [143]. Additionally, Keggin-type PMo_12_V_3_ achieved 99.3% MB removal after 65 min of UV irradiation [53], while Keggin-type PMo_11_V obtained 50.8% MB removal efficiency after 120 min of visible light exposure [56]. These findings underscore that the composition and structure of POMs are pivotal in governing their photocatalytic activity.

### 3.2. Enhancement Strategies for POM-Based Materials

By employing strategic material combinations and thoughtful interface design, it can not only to augment the specific surface area of POM-based materials but also to enhance the interaction between substrates and active sites. Additionally, effective electron–hole separation can be achieved, resulting in a significant improvement in photocatalytic efficiency. In accordance with the functional mechanism, the primary strategies for achieving this enhancement are outlined as follows:

(1) Incorporation onto Suitable Carriers: The introduction of POMs into appropriate carriers, such as SiO_2_, provides a stable scaffold that enhances stability and activity [126,138,144];
(2) Surface Modification: After immobilizing POMs molecules onto carrier surfaces, the introduction of light-absorbing groups or the adjustment of surface chemistry enhances the POMs’ absorption capacity, even within the visible light range [66,67,83];
(3) Fabrication of Composite Materials: The combination of POMs with other optically active materials, such as semiconductor nanoparticles (TiO_2_ and ZnO), results in synergistic composite systems that elevate visible light absorption and electron transfer efficiency [95,98,106];
(4) Introduction of Conjugated Structures: The incorporation of conjugated structures, such as benzene rings or carbazole moieties, into the POMs framework extends its light-absorption range, enabling broader visible light utilization [145];
(5) Nano materialization: The transformation of POMs molecules into nanoparticles increases the surface area, thereby enhancing light absorption efficiency [119,124].

POM-based materials’ photocatalytic activity and stability depend on both compositions and synthesis methods. Common methods include the following:

(1) Dipping: Simple but may result in uneven impregnation, especially for large materials.
(2) Grinding: Easy but challenging to control particle size, leading to non-uniformity of materials.
(3) Heating Reflux: Versatile for various reactions, offering precise temperature control but requiring reflux equipment and involving intricate procedures.
(4) One-Pot Synthesis: Suitable for complex multi-component materials, saving time and resources by avoiding intermediate steps, but necessitates precise reaction control and may produce byproducts.
(5) Hydrothermal and Solvothermal Methods: Ideal for synthesizing crystals, nanoparticles, and complex structures, with control over material size and shape. However, they typically involve high-temperature and high-pressure conditions.

The choice of the most suitable synthesis method depends on the desired material properties and the specific experimental conditions.

## 4. Application Potential of POM-Based Materials

Factors such as intricate synthesis, purity requirements, and material specificity may result in higher costs for POM-based photocatalysts compared to simpler counterparts. However, in situations where catalytic activity, selectivity, and long-term stability are pivotal considerations, the performance advantages and potential long-term benefits of POMs position them as the preferred choice for photocatalysts. These advantages can be assessed by researchers and industries to ascertain the cost-effectiveness of utilizing POM-based materials in particular applications.

## 5. Conclusions and Perspectives

The utilization of POM-based photocatalysis presents a promising avenue for addressing water contamination issues through effective dye degradation. The distinctive optical properties, diverse catalytic sites, and adaptable energy levels inherent to POMs offer a compelling platform for efficient solar-driven catalysis across a broad spectrum, including visible light. The presence of metal–oxygen clusters and vacant d orbitals within transition metals facilitates electron transitions from oxygen to metals upon light exposure, resulting in the generation of electron–hole pairs. These pairs serve in the direct oxidation of dyes or react with H_2_O/O_2_ to produce ^•^OH/O_2_^•−^ and other oxidative species.

The synergy achieved through the coupling of POMs with other materials in heterojunctions enhances light absorption and photocatalytic efficiency. Notably, the adjustment of band structures and interface electronic states within heterojunctions facilitates improved electron and hole migration, thereby reducing charges recombination and bolstering the photocatalytic efficiency. Beyond the realm of heterojunctions, the integration of POMs with materials like TiO_2_ and organic-inorganic hybrid frameworks like MOFOF and COFs holds promise for enhancing light-absorption efficiency and catalytic activity. Strategic approaches such as carrier incorporation, surface modification, composite material fabrication, the introduction of conjugated structures, and nanomaterialization contribute to the amplification of light utilization and efficiency.

As the field progresses, future research necessitates more experimental investigations and the development of theoretical models. A thorough exploration into the generation and action of ROS is essential to uncover the precise mechanism of dye degradation. Furthermore, as the photocatalytic performance advances, additional research may be required to clarify the precise relationship between the composition, structure, and photocatalytic activity of POMs. Additionally, translating laboratory successes into practical applications becomes crucial. Scalable synthesis methods, material stability assessment under real conditions, and effective integration into water treatment systems are paramount. Efficient and practical POM-based water remediation demands interdisciplinary collaboration and a deep understanding of materials science, catalysis, and environmental engineering. By tackling challenges and seizing opportunities, POM-based photocatalysis can significantly influence the advancement of water purification technologies.

## Data Availability

Not applicable.
